# Chronic Plaque Psoriasis as a Predictor of Spinal Osteoproliferation on Magnetic Resonance Imaging (MRI)

**DOI:** 10.7759/cureus.106529

**Published:** 2026-04-06

**Authors:** Kristen Tamsil, Madison Gackle, Srujani Das, Raisa Suha, Zarin Kothari, Simran Agarwal, John T Schwartz

**Affiliations:** 1 Osteopathic Medicine, Touro University College of Osteopathic Medicine, Vallejo, USA; 2 Orthopaedic Surgery, Arizona College of Osteopathic Medicine, Glendale, USA; 3 Orthopaedic Surgery, William Carey University College of Osteopathic Medicine, Hattiesburg, USA; 4 Medicine, Stony Brook University, Stony Brook, USA; 5 Orthopaedic Surgery, Wayne State University School of Medicine, Detroit, USA; 6 Orthopedic Surgery, Ross University School of Medicine, Barbados, USA; 7 Orthopedic Surgery, Valley Consortium for Medical Education, Modesto, USA

**Keywords:** axial spondyloarthritis, dermatology, enthesophyte, magnetic resonance (mr), mri, orthopaedics, osteoproliferation, psoriasis, rheumatology

## Abstract

Chronic plaque psoriasis is a systemic inflammatory condition that extends beyond the skin and has been linked to musculoskeletal changes, including excessive bone formation along the spine. Emerging evidence indicates that individuals with psoriasis are more likely to demonstrate spinal bony overgrowth than those without the disease. Although biologically plausible mechanisms have been proposed to explain this relationship, there is currently no clear framework for translating the association into clinical decision-making. Existing practice recommendations do not specify which patients might benefit from spinal magnetic resonance imaging (MRI) evaluation, nor do they clarify whether identifying early, asymptomatic changes influences long-term outcomes. The routine use of MRI is further complicated by its expense, variability in imaging techniques, and the need for adequate clinical suspicion before testing. As a result, its role in standard psoriasis management remains ill-defined. Further investigation is needed to establish uniform imaging criteria, identify high-risk patient subgroups, and assess outcomes over time to determine whether earlier identification of spinal involvement can alter disease trajectories. For now, the value of this review lies in drawing attention to the observed relationship between psoriasis and spinal bone formation, while emphasizing the substantial gaps that must be addressed before this knowledge can inform evidence-based screening or management approaches.

## Introduction and background

Introduction

Chronic plaque psoriasis is a common systemic inflammatory skin disorder with well-documented associations with psoriatic arthritis (PsA), cardiometabolic disease, and mood disorders [1]. However, beyond its cutaneous and peripheral joint involvement, an emerging area of interest is its link to axial musculoskeletal pathology, specifically spinal osteoproliferation. Spinal osteoproliferation, which includes syndesmophyte formation, enthesophytes, and paravertebral ossification, can result in stiffness, pain, and irreversible spinal deformity [2]. Despite its potential to cause lasting disability, axial disease in patients with psoriasis often goes unrecognized until late stages, especially in patients without overt psoriatic arthritis. 

This review examines whether cutaneous psoriasis can serve as an early indicator for silent axial disease. Current data suggest that systemic inflammation inherent to psoriasis could contribute to osteoproliferative changes in the spine via shared immunopathogenic mechanisms, such as interleukin (IL)-23/IL-17-driven entheseal inflammation and aberrant bone remodeling [3-5]. Notably, these changes may occur even in the presence of normal bone mineral density (BMD), challenging traditional paradigms for skeletal screening and risk assessment. 

While skin-only psoriasis is typically managed with topical therapies, phototherapy, or non-biologic systemic agents, axial manifestations warrant early initiation of biologic or targeted disease-modifying antirheumatic drugs (DMARDs) to prevent irreversible structural progression [5-7]. Identifying axial involvement earlier, particularly through targeted magnetic resonance imaging (MRI) in appropriate psoriasis patients, could shift patients onto this arthritis-level treatment pathway sooner, potentially altering the disease course and preserving long-term spinal function. This paper aims to evaluate the evidence linking chronic plaque psoriasis to spinal osteoproliferation and to determine whether earlier recognition of this association could improve long-term outcomes.

Methods

A comprehensive literature review was conducted to examine current evidence related to chronic plaque psoriasis and axial musculoskeletal involvement. The potential mechanisms, clinical implications, and imaging characteristics associated with spinal osteoproliferation and psoriasis were evaluated. Relevant studies were identified through a focused search of PubMed, Google Scholar, and other scientific databases. Key search terms included combinations of “psoriasis,” “psoriatic arthritis,” “axial disease,” “spondyloarthritis,” “osteoproliferation,” “enthesitis,” “IL-17,” and “IL-23,” as well as combinations of these terms. The search conducted by the authors was restricted to peer-reviewed journal articles, clinical trials, literature reviews, and meta-analyses published in English. No formal risk-of-bias assessment or quantitative synthesis was performed. Much of the existing literature consists of observational and cross-sectional studies with variability in imaging technique, lesion definitions, and scoring systems. These factors limit direct comparison across studies. The purpose of this review is to synthesize the evolving body of literature addressing axial pathology in psoriasis, while accounting for diagnostic complexity. This review aims to distinguish emerging concepts from established evidence and underscore priorities for future research.

## Review

Pathophysiology

Immunologic Mechanisms in Psoriasis 

Psoriasis, once believed to be exclusively related to the skin disease, has now been recognized as a systemic immunologic disease [6]. Clinically, cutaneous psoriasis most often affects the elbows, knees, scalp, and lower back, and can be complicated by inflammatory arthritis [7]. Its pathogenesis reflects a complex interplay between the innate and adaptive immune systems, with pro-inflammatory cytokines, particularly tumor necrosis factor (TNF)-α, playing central roles. Two distinct phases characterize disease development: initiation and maintenance [8]. Keratinocytes, found in the epidermis, play a key role in the pathogenesis of psoriasis. In the initiation phase, epidermal injury triggers keratinocytes to release signaling molecules that activate plasmacytoid dendritic cells (pDCs), leading to the release of TNF-α and other cytokines [8, 9]. These mediators stimulate myeloid dendritic cells (mDCs) to migrate to regional lymph nodes, where they secrete cytokines that drive naïve T-cell proliferation and differentiation into Th1, Th17, and Th22 subsets [8, 9]. In the IL-23/Th17 axis, Th17 cells produce IL-17A/F and IL-22, which promote keratinocyte hyperproliferation and sustain the psoriatic phenotype, thus facilitating entrance into the maintenance phase [8, 10]. This persistent IL-23/IL-17-driven inflammation underlies not only cutaneous features of psoriasis but also its systemic associations, including spondyloarthritis (SpA), providing a mechanistic basis for potential axial involvement even in skin-only disease.

Mechanisms of Osteoproliferation and Proposed Shared Inflammatory Pathways

Osteoproliferation results from dysregulation of tightly balanced bone remodeling pathways [11]. Osteoblasts, derived from mesenchymal stem cells (MSCs), are regulated by pro-osteogenic signals such as bone morphogenetic proteins (BMPs) and Wnt pathways [12]. Overactivation of these pathways promotes abnormal bone formation, particularly in the spine. In the Wnt pathway, Wnt3a enhances osteoblast differentiation, proliferation, and survival, and experimental animal models of spondyloarthritis support the importance of Wnt signaling in the osteoproliferative pathways underlying spondyloarthritis [2, 13]. These pro-osteogenetic changes are closely linked to inflammation.

IL-17 is a key driver of psoriatic inflammation [14-17] and participates directly in entheseal remodeling in spondyloarthritis [10, 14, 15]. Wnt signaling interacts with IL-17A in a cell context-dependent manner, influencing both inflammatory and osteogenic processes [18]. Wnt5a is upregulated in psoriatic lesions, implicating it in disease susceptibility and paralleling its role in spinal arthropathy development [7]. Although a direct connection between Wnt3a and psoriasis has not been fully established, the shared IL-17-Wnt axis provides a biologically plausible link between chronic skin inflammation and spinal osteoproliferation [19]. The overlap between IL-17-driven inflammation and Wnt-mediated bone remodeling suggests that cutaneous psoriasis could serve as an early signal for underlying axial skeletal disease. Recognizing these mechanisms strengthens the rationale for targeted musculoskeletal assessment in high-risk patients. 

Clinical manifestations and musculoskeletal involvement

Cutaneous Features of Chronic Plaque Psoriasis

Chronic plaque psoriasis, also known as psoriasis vulgaris, manifests as sharply demarcated, erythematous plaques with an overlying silvery-white scale most commonly affecting the scalp, extensor surfaces such as the elbows and knees, and the lumbosacral region [20]. Painful fissures may occur on the palms and soles. The scaling results from epidermal hyperproliferation, and removal of these plaques reveals pinpoint bleeding [21]. It is estimated that 40-50% of patients with cutaneous psoriasis exhibit nail findings during the course of their disease, while the rate in patients with psoriatic arthritis is significantly higher, ranging from 80-90% [22, 23]. When evaluating cutaneous features of psoriasis, dermatologists should carefully assess for nail changes such as pitting, nail plate crumbling, leukonychia, red spotting, onycholysis, and hyperkeratosis [24]. Nail involvement, especially matrix-related changes such as pitting and onycholysis, is observed in the majority of patients with PsA and represents one of the most robust clinical predictors of transition from cutaneous psoriasis to inflammatory arthritis [22, 25]. Rouzoud et al. suggested in their meta-analysis that psoriasis patients with nail involvement have nearly a threefold increased risk of PsA [odds ratio (OR) 2.92, 95% confidence interval (CI) 2.34-3.64], with onycholysis associated with more than double the odds of PsA (OR 2.38, 95% CI 1.74-3.26) [25]. Taken together, nail changes are not only a visible marker of disease severity but also may be a clinically meaningful predictor of musculoskeletal progression, underscoring the importance of earlier recognition. 

Musculoskeletal Involvement in Psoriasis: Overview

Musculoskeletal manifestations of psoriasis extend beyond the well-known skin and joint manifestations of psoriatic arthritis, encompassing early changes in the peripheral joints, entheses, and axial skeleton. While PsA develops in approximately 30% of psoriasis patients, often years following cutaneous onset [26], growing evidence suggests that inflammation and bone remodeling can occur even in the absence of clinically apparent arthritis. This subclinical inflammation can often be detected upon imaging using ultrasound or MRI. In a study conducted by Zuliani et al., 40 patients with psoriasis and no musculoskeletal symptoms were compared to 20 healthy controls. Ultrasound revealed active synovitis in 27.5% and active enthesitis in 20% of asymptomatic psoriasis patients, compared to 0% in healthy controls [27], indicating that the disease process often precedes clinical diagnosis. Patients with arthralgia and positive imaging findings may represent a preclinical stage with significantly increased risk of progression to PsA [28]. In one prospective study, individuals with MRI-confirmed subclinical synovitis and joint symptoms had a 60% likelihood of developing PsA, compared to only 13% in asymptomatic patients with negative MRI findings [29]. Together, these findings suggest that relying on symptoms alone risks missing a window for intervention. At present, there is limited direct evidence that treating preclinical axial involvement results in superior outcomes compared with treatment initiated after symptom onset; however, studies showing that subclinical inflammatory findings are associated with higher rates of progression to clinically apparent PsA support the rationale for early identification and close monitoring [29]. Incorporating targeted imaging into the assessment of high-risk psoriasis patients may aid in earlier identification of musculoskeletal involvement, although current evidence remains insufficient to conclude that this approach definitively alters long-term structural outcomes or treatment trajectories. 

Imaging evaluation and therapeutic considerations in psoriatic spinal disease

MRI as the Key Diagnostic Modality

MRI is the most sensitive modality for early detection of osteoproliferative changes in the spine associated with chronic plaque psoriasis [30]. It visualizes both osseous and soft tissue abnormalities, including syndesmophytes, enthesophytes, and paravertebral ossifications, as well as inflammatory features such as bone marrow edema and enthesitis, which frequently precede structural changes [31]. Fat-suppressed T2-weighted or Short Tau Inversion Recovery (STIR) sequences are optimal for early diagnosis [30]. Preoperatively, MRI is essential for evaluating degenerative disc disease, facet joint instability, spinal canal stenosis, and foraminal narrowing, findings that are critical for surgical planning. Identifying axial disease in psoriasis patients via MRI is not only diagnostic, but can also change the treatment trajectory from skin-focused therapy to systemic biologics or DMARDs, a shift with potential to alter long-term outcomes. 

Clinical Implications: Treatment Divergence and the Rationale for Early Imaging

Management of chronic plaque psoriasis differs fundamentally from that of psoriatic arthritis (PsA), and this divergence becomes critical when imaging demonstrates axial or osteoproliferative involvement. First-line therapy for skin-only psoriasis includes topical agents, phototherapy, or nonbiologic systemic therapies for moderate to severe skin disease [32]. In contrast, axial PsA, especially with evidence of structural progression, requires the immediate onset of systemic treatment to prevent joint damage, with biologic or targeted synthetic DMARDs [33]. Patients may present with nonspecific back pain or stiffness and show evidence of subclinical inflammation on MRI. These early imaging findings can be the first indication of evolving axial PsA and may represent a critical window for earlier recognition and intervention [34]. For this reason, the American Academy of Dermatology and National Psoriasis Foundation recommend PsA screening at every dermatologic visit to avoid delayed diagnosis and irreversible joint damage [32, 35]. MRI might also detect subclinical enthesitis or synovitis, which are predictive of progression and support early referral to rheumatology for further evaluation [36, 37]. While routine screening for degenerative spinal disease in psoriasis is not recommended, guidelines emphasize maintaining a high index of suspicion for axial PsA in younger patients with unexplained spinal symptoms [38, 39]. Identifying these on imaging not only helps with early diagnosis but also provides an opportunity to refine treatment strategies, minimize disability, and avoid irreversible spinal damage. This potential to alter the treatment trajectory is the central rationale for more proactive, targeted MRI screening in select high-risk psoriasis patients. 

Evidence linking psoriasis and spinal osteoproliferation

Summary of Existing Studies and Their Methodologies

MRI has emerged as a powerful tool to detect subclinical axial disease in psoriatic populations, even before clinical psoriatic arthritis (PsA) manifests. Bratu et al. (2019) performed whole‑spine and sacroiliac joint MRI using STIR sequences in patients with cutaneous psoriasis and without clinical arthritis. They reported minimal inflammation or structural changes, suggesting that routine imaging in skin-only psoriasis may not be justified [40]. Crucially, this also highlights that even highly sensitive imaging may only detect axial disease in a minority of asymptomatic patients-an important insight for dermatologists evaluating back pain.

In contrast, broader literature demonstrates that careful use of advanced MRI protocols, especially STIR and fat-suppressed T2 sequences, enhances detection of subtle inflammatory signs such as bone marrow edema and early enthesitis [26, 41]. Crespo-Rodriguez et al. affirm that these sequences significantly improve sensitivity in early or subclinical disease, while McQueen et al. showed that syndesmophytes and inflammation can be detected before overt arthritis. These findings are supported by international expert consensus, including Assessment of SpondyloArthritis International Society (ASAS)/ Outcome Measures in Rheumatology (OMERACT) and Ostergaard et al., which recommend whole-spine MRI protocols using fat-suppressed and STIR imaging to reliably detect both inflammatory and chronic structural lesions in psoriatic disease [37]. Together, these data indicate that while axial ossification is not pervasive among all psoriasis patients, it may be selectively present, especially in individuals with long-standing or severe skin disease. Although methodologies differ, consistent MRI findings across psoriatic cohorts support the concept that silent axial involvement may occur even in the absence of clinical arthritis. Early identification of these lesions may have prognostic value, especially in patients with high skin burden or known risk factors for PsA development. 

Prevalence of Osteoproliferation in Psoriasis Patients Without Psoriatic Arthritis

The retrospective analysis by Diaz et al. assessed whole‑spine and sacroiliac joint (SIJ) MRI in 93 patients with either psoriasis or PsA to characterize the axial involvement. Among the 28 patients with psoriasis alone, MRI-defined axial spondyloarthritis (MRI-SpA) was detected in 9.7% using ASAS criteria and 12.9% by radiologist impression, despite back pain being present in 81.7% of the overall cohort [42]. Notably, approximately 25% of MRI-SpA cases showed isolated spondylitis without accompanying sacroiliitis, highlighting that structural spinal changes may occur independently of SIJ involvement [42]. In addition, MRI in this study was not performed routinely but rather based on clinical suspicion, often due to reported back pain, highlighting that while back pain was a commonly reported symptom, it did not reliably predict the presence of MRI-defined disease. 

This low prevalence of MRI-detected disease in psoriasis-only individuals suggests that spinal osteoproliferation is relatively uncommon in the absence of clinically evident PsA. In contrast, larger PsA cohorts such as the Psoriatic Arthritis Study (PsSpA) by Jadon et al. in 2017, have reported syndesmophytes in up to 33-40% of patients with axial PsA, including a substantial subset with isolated spondylitis [43]. The contrast between these findings and the low MRI-SpA rate observed by Diaz et al., in psoriasis-only patients, reinforces the notion that cutaneous psoriasis without PsA rarely presents with axial structural lesions on imaging. 

Although spinal involvement appears rare overall in psoriasis-only individuals, certain factors may help identify the affected subset. Diaz et al. reported that patients with radiologist-confirmed MRI-SpA were more often male (83.3% vs 42%) and had a shorter duration of psoriasis (5.8 vs 14.7 years) compared with those without MRI-SpA [42]. In regression analyses, only male sex remained significantly associated with MRI-SpA (OR 6.91) [42]. These findings suggest that demographic factors may influence the likelihood of detecting MRI-SpA, but the strength of the evidence remains limited. Moreover, the specificity of MRI warrants caution. Degenerative and age-related changes are frequently observed, raising the possibility that some findings labeled as SpA may be incidental. Taken together, current data support an association between psoriasis and MRI features of axial disease, but fall short of establishing causality or providing a framework for clinical application. Further research is needed to clarify the natural history of these imaging findings and determine whether they truly identify patients at risk for meaningful progression. These findings support a targeted approach to imaging, in which clinical symptoms, rather than routine screening, guide MRI acquisition, which is consistent with standard practice in the evaluation of axial disease. 

Impact of Psoriasis Severity and Duration on Axial Musculoskeletal Prognosis

Evidence suggests that both the severity and chronicity of psoriasis may be linked to axial skeletal changes, even in patients without a diagnosis of psoriatic arthritis. Poddubnyy et al. observed that patients with long-standing psoriasis often had subclinical inflammatory lesions in the spine and sacroiliac joints, raising the possibility that cumulative inflammatory burden may affect axial structures over time [44]. Similarly, Diaz et al. reported that higher PASI scores and longer disease duration were associated with structural changes on MRI, including syndesmophytes and enthesophytes, even in the absence of PsA classification [42]. These findings suggest there may be a subgroup of psoriasis patients with a preclinical axial phenotype, although the strength of this evidence remains limited. 

Interestingly, mechanistic data from animal studies support a biological rationale, as chronic activation of the IL-23/IL-17 pathway has been shown to drive entheseal inflammation and osteoproliferation [45]. At present, these findings should be viewed as preliminary. MRI abnormalities are not specific, and degenerative or incidental changes are common, particularly in older patients. The available studies are small, observational, and heterogeneous, limiting confidence in the strength of the associations. Current data therefore support a possible link between psoriasis severity, duration, and axial changes, but fall short of demonstrating causality or predictive value. Larger, prospective studies are needed to clarify whether these imaging findings identify patients at genuine risk for progression and whether they have practical utility in guiding clinical care. 

Clinical implications

Surgical and Medical Management of Axial Disease

Spinal osteoproliferation in patients with psoriasis can lead to stiffness, pain with movement, and functional limitations that reduce quality of life [46]. In some cases, when the sacroiliac (SI) joint is affected, SI joint injections are used for symptom relief, and SI joint fusion procedures may be considered when symptoms are severe or refractory [47]. Compared to those with peripheral disease alone, patients with axial psoriatic arthritis tend to report worse physical function, higher rates of work disability, and lower quality of life scores [47]. Neurologic complications from stenosis or instability, such as paresthesias, gait disturbance, or myelopathy, represent advanced disease, where surgical decompression or fusion may be required [47]. However, robust data specific to spinal surgery in axial PsA are lacking. A population-based cohort by Nystad et al. found that over 20% of patients with psoriatic arthritis underwent at least one musculoskeletal surgery over the course of their disease, including joint replacement, arthroscopies, and tendon surgeries, but spine-specific outcomes were not reported [48]. These gaps highlight the need for more focused data on the surgical management of axial disease in this population. 

On the medical side, biologic therapies remain the mainstay of treatment. TNF inhibitors (e.g., infliximab, etanercept) and Janus kinase (JAK) inhibitors (e.g., tofacitinib, upadacitinib) have shown efficacy in reducing inflammation and improving symptoms, specifically in the context of axial disease [49-51]. Observational evidence suggests that earlier initiation may reduce progression, though most trials to date lack long-term structural outcomes in axial PsA [49], and the true impact of biologic therapy on preventing irreversible spinal damage is still uncertain. Other biologics targeting the IL-17 and IL-23 pathways, which are both implicated in the pathological inflammation seen with axial disease, have been evaluated for efficacy as well. IL-17A inhibitors (e.g., secukinumab, ixekizumab) have been found to have significantly greater response rates compared to placebo [51]. IL-12/23 inhibitors (e.g., ustekinumab) and IL-23 inhibitors (e.g., guselkumab) still have insufficient evidence for use in axial disease [51]. Implementation of management should be made based on prior history with biologics, with literature varying based on certain subsets of axial or cutaneous disease patients [51]. More rigorous studies are needed to determine whether early recognition and treatment alter the natural history of spinal disease in this setting, as well as evaluation in the overlap of disease progression 

Implications for Interdisciplinary Screening and Management

Dermatologists are the primary point of contact and often the only specialists who manage patients with psoriasis, making them well placed to recognize early musculoskeletal symptoms. Estimates suggest that up to two-thirds of patients with psoriatic arthritis develop axial disease with associated loss of function [44]. While the strength of this evidence is variable, these findings highlight the importance of including musculoskeletal history and symptom review as a part of routine dermatologic care. Simple questions about back pain, morning stiffness, or fatigue can increase the likelihood of identifying patients who may benefit from referral. Screening questionnaires such as the Psoriasis Epidemiology Screening Tool (PEST) or the Early Arthritis for Psoriatic Patients (EARP) may support this process [52, 53], though their use in clinical practice remains inconsistent. 

For patients with possible neurologic involvement, instruments such as the dropping, off-balance, weakness, numbness (DOWN) questionnaire and neck disability index (NDI) can help flag symptoms of myelopathy or radiculopathy [54, 55]. In practice, however, time constraints and limited formal musculoskeletal training often preclude dermatologists from using these tools regularly. Incorporating a brief musculoskeletal and neurological history into standard intake forms could offer a more consistent approach without significantly extending visits. At present, these strategies are best viewed as pragmatic aids rather than proven interventions. More evidence is needed to determine whether earlier recognition meaningfully alters outcomes, but encouraging interdisciplinary awareness remains a reasonable step toward more comprehensive care. 

Future directions

Gaps in Current Knowledge

Current studies linking psoriasis and spinal osteoproliferation are largely cross-sectional or retrospective, limiting our ability to establish causation or track longitudinal progression of disease [56-60]. Moreover, heterogeneity in MRI acquisition protocols, such as magnetic field strength, spinal levels, and lesion scoring systems, introduces variability that undermines diagnostic reliability [37]. Although standardized definitions (e.g., ASA, CanDen) have been proposed to assist in the recognition of axial involvement, their application remains inconsistent and, in practice, somewhat restrictive [57]. While Th17/IL-23-driven inflammation has been implicated in entheseal ossification, the precise molecular cascade linking cutaneous and skeletal pathology remains poorly understood [56-58]. Importantly, few investigations stratify by psoriasis phenotype and duration of disease, leaving uncertainty as to which patients are at greatest risk. Addressing these methodological limitations is essential for generating clinically meaningful evidence that can guide management of the systemic consequences of psoriasis. 

Recommendations for Future Research

To improve diagnostic consistency and comparability, future studies should evaluate new patients using standardized MRI protocols with harmonized sequences and scoring systems such as CanDen and ASAS [37]. Longitudinal, prospective designs with serial imaging and clinical assessment are particularly important to clarify the natural history of osteoproliferation and explore potential causal relationships in chronic plaque psoriasis [56]. Pilot work using contrast-enhanced and vascular-sensitive imaging techniques may help visualize early inflammatory changes before structural damage occurs [36]. Stratifying participants by phenotype, disease severity, and musculoskeletal symptoms may also help identify subgroups at greater risk for axial involvement [59] while inclusion of asymptomatic patients could provide insight into subclinical disease and its prognostic value [40]. Incorporating patient-reported outcomes alongside imaging measures is essential for linking radiographic findings to clinically meaningful endpoints. Finally, the use of uniform criteria and multicenter collaboration would enhance generalizability and allow for higher-level evidence synthesis. 

Potential for Predictive Screening Guidelines and Predictive Models

These results suggest the potential to build predictive screening models and follow patients with chronic plaque psoriasis at risk for spinal osteoproliferation. Currently, the predictive models for PsA rely on risk factors such as chronological age, sex, BMI, and treatment history, without taking into account axial disease or spinal imaging variables [60]. The most challenging issue hampering progress is the lack of a shared standard because of disparate imaging terms, including definitions and protocols, which could be addressed by a new dataset [36]. Current evaluations employing cross-sectional data sets are not able to monitor disease progression or develop predictive models over time [61]. By adding genetic and molecular markers, such as from blood and urine samples, as well as circulating genetic sequences to our models, we may improve sensitivity and stratification as a whole. These tools can also help create treatments and methods that offer better, earlier interventions for patients with psoriatic arthritis, and hence more tailored pathways. Lastly, although there are recommendations for psoriatic arthritis screening, no such recommendations are available for spinal disease, further indicating the need for additional clinical consensus and screening guidelines.

## Conclusions

This review highlights a growing body of evidence linking chronic plaque psoriasis to spinal osteoproliferative changes, including enthesophytes, syndesmophytes, and paravertebral ossifications. Although classically considered a cutaneous disease, psoriasis demonstrates systemic inflammatory behavior with potential musculoskeletal consequences that may precede or occur independently of psoriatic arthritis. Our findings suggest that spinal involvement may occur subclinically in a meaningful subset of patients, particularly those with severe or longstanding skin disease. However, the pathway to identifying this subset and intervening in a practical, cost-effective manner remains elusive. While psoriasis and spinal disease appear linked, the evidence base has not yet matured enough to establish practical strategies for screening or intervention, particularly with the overall high cost of MRI. Future research must focus on defining thresholds of clinical suspicion, validating standardized imaging protocols, and demonstrating that early detection translates into meaningful long-term outcomes. Until then, this paper serves primarily to call attention to the gap between emerging biologic plausibility and actionable clinical pathways, highlighting the need for continued interdisciplinary collaboration to bridge that divide. 
